# The substrate tolerance of alcohol oxidases

**DOI:** 10.1007/s00253-015-6699-6

**Published:** 2015-07-08

**Authors:** Mathias Pickl, Michael Fuchs, Silvia M. Glueck, Kurt Faber

**Affiliations:** Austrian Centre of Industrial Biotechnology (ACIB GmbH), Petersgasse 14, 8010 Graz, Austria; Department of Chemistry, Organic & Bioorganic Chemistry, University of Graz, Heinrichstrasse 28, A-8010 Graz, Austria

**Keywords:** Oxidation, Biocatalysis, Alcohol oxidase, Substrate tolerance, Flavoprotein, Cu-containing oxidase

## Abstract

Alcohols are a rich source of compounds from renewable sources, but they have to be activated in order to allow the modification of their carbon backbone. The latter can be achieved via oxidation to the corresponding aldehydes or ketones. As an alternative to (thermodynamically disfavoured) nicotinamide-dependent alcohol dehydrogenases, alcohol oxidases make use of molecular oxygen but their application is under-represented in synthetic biotransformations. In this review, the mechanism of copper-containing and flavoprotein alcohol oxidases is discussed in view of their ability to accept electronically activated or non-activated alcohols and their propensity towards *over-oxidation* of aldehydes yielding carboxylic acids. In order to facilitate the selection of the optimal enzyme for a given biocatalytic application, the substrate tolerance of alcohol oxidases is compiled and discussed: Substrates are classified into groups (non-activated *prim*- and *sec*-alcohols; activated allylic, cinnamic and benzylic alcohols; hydroxy acids; sugar alcohols; nucleotide alcohols; sterols) together with suitable alcohol oxidases, their microbial source, relative activities and (stereo)selectivities.

**Electronic supplementary material:**

The online version of this article (doi:10.1007/s00253-015-6699-6) contains supplementary material, which is available to authorized users.

## Introduction

Oxidation represents a fundamental reaction in nature (Hollmann et al. 2011; Turner 2011), and oxidases are a prominent subclass of redox enzymes, which use oxygen either as oxidant or as electron acceptor. This property made them particularly attractive for the production of chemicals (Vennestrom et al. 2010). In this context, the oxidation of alcohols is an important transformation in synthetic chemistry, which allows to introduce carbonyl groups, which represent excellent acceptors for *C*-, *N*-, *O*- and *S*-nucleophiles and thereby allows the extension of a given carbon backbone. Consequently, a large number of protocols has been developed, which depend on (i) transition metals in stoichiometric (e.g. Cr, Mn) or catalytic amounts (e.g. Ru, Fe), (ii) metal-free oxidations according to Swern or Pfitzner-Moffat (Pfitzner and Moffatt 1963; Omura and Swern 1978), (iii) molecular oxygen as oxidant (Tojo and Fernández 2006) and more recently, (iv) organocatalysts, such as TEMPO (Wertz and Studer 2013).

**Scheme 1:**
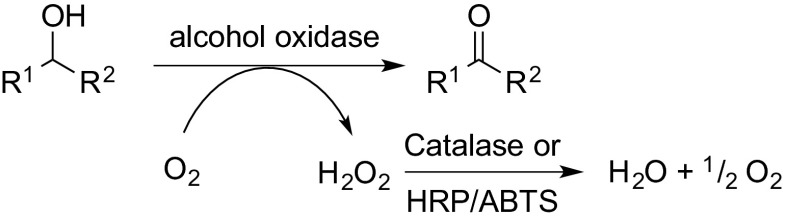
Biocatalytic oxidation of alcohols using an alcohol oxidase

In a related fashion, alcohol oxidases convert primary and secondary alcohols to aldehydes and ketones, respectively. During this reaction, molecular oxygen is reduced to hydrogen peroxide. In order to avoid enzyme deactivation, a catalase is usually employed, particularly on preparative scale. For screening purposes, a spectrophotometric assay based on horse radish peroxidase (HRP) together with a suitable artificial electron acceptor, such as 2,2′-azino-bis(3-ethylbenzthiazoline-6-sulfonic acid) (ABTS) may be employed (Scheme 1). The ABTS-radical generated shifts its absorption maximum (Baron et al. 1994; Uwajima and Terada 1980).

**Scheme 2:**
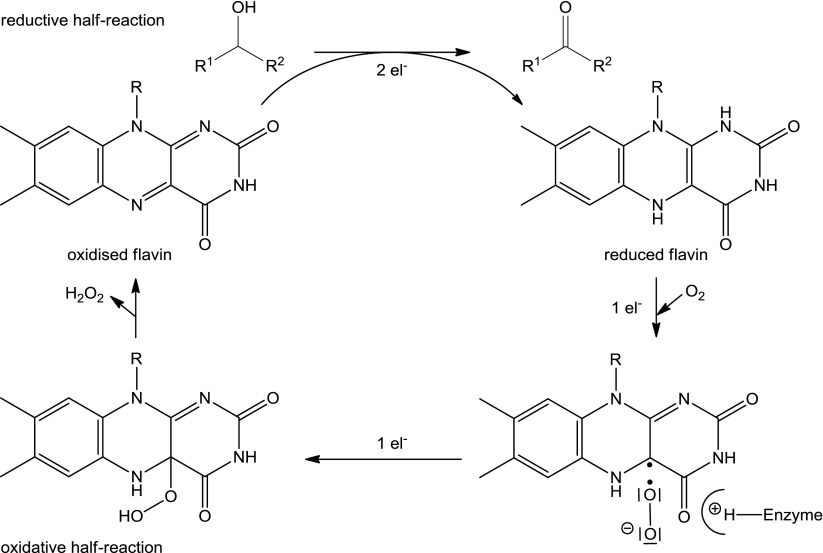
Catalytic cycle of flavin-containing alcohol oxidases

Although cofactor-lacking oxidases are reported (Fetzner and Steiner 2010), commonly used alcohol oxidases depend on flavin (Macheroux et al. 2011; Dijkman et al. 2013) or a metal (Cu) as a cofactor (Whittaker 2003), which mediates the electron transfer. In flavoprotein oxidases, the oxidation proceeds via two half reactions, where the alcohol is first oxidised by a two-electron transfer during the *reductive half reaction*, yielding reduced flavin. The oxidised flavin is regenerated by a stepwise single-electron transfer via the *oxidative half reaction*, which requires triplet oxygen, as it is a spin-forbidden reaction. Hence, di-oxygen acts as single-electron acceptor and forms superoxide (O_2_^−·^), stabilised by a positively charged histidine residue (Dijkman et al. 2013; Wongnate et al. 2014). Another single-electron transfer yields a covalent hydroperoxy flavin intermediate, which eliminates hydrogen peroxide and re-forms oxidised flavin (Scheme 2) (Gadda 2012). The highly unstable C_4_a-hydroperoxyflavin intermediate has only been detected for pyranose oxidase (P2O) (Mattevi 2006; Chaiyen et al. 2012; Wongnate and Chaiyen 2013).

**Scheme 3:**
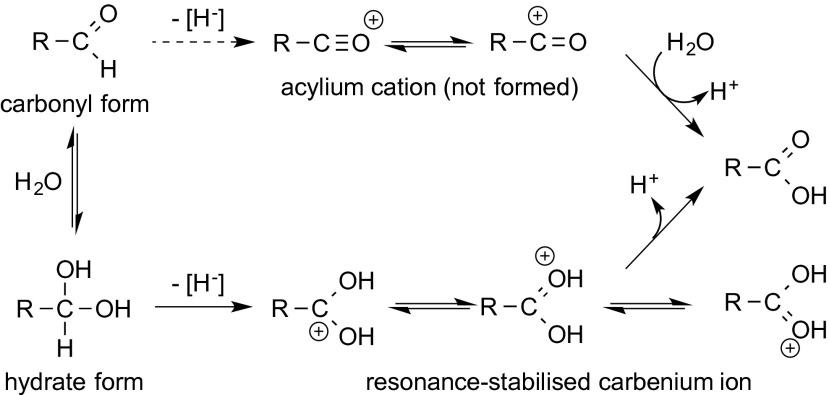
Aldehyde oxidation via the hydrate intermediate

The oxidation of primary alcohols catalysed by flavoprotein oxidases does not necessarily stop at the aldehyde stage, but may further proceed to the corresponding carboxylic acid. This second oxidation step is mechanistically less investigated, but it is obvious that the actual substrate is the aldehyde hydrate (*gem*-diol), rather than its carbonyl form, because hydride abstraction from the former yields a doubly resonance-stabilised oxocarbenium cation, which upon expulsion of H^+^ furnishes the carboxylic acid. In contrast, hydride abstraction from the carbonyl form would lead to a highly unstable (hypothetical) acylium cation, which would be quenched by a water molecule (Scheme 3).

**Scheme 4:**
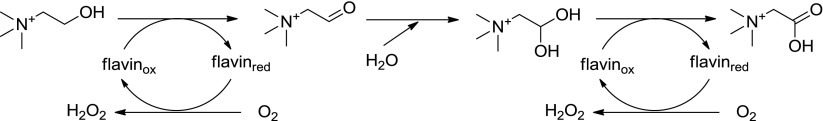
Two-step oxidation of choline by choline oxidase yielding betaine

This mechanism for over-oxidation has been proposed for choline oxidase (CHO), whose natural role is the formation of *N*-trimethylammonium glycine (‘betaine’) from choline via the aldehyde hydrate through a two-step oxidation (Scheme 4) (Rungsrisuriyachai and Gadda 2008).

The over-oxidation of alcohols to carboxylic acids has been observed not only for choline oxidase but also for other flavoprotein oxidases, such as alditol oxidase (AldO), aryl alcohol oxidase (AAO), hydroxymethyl furfuryl oxidase (HMFO), hexose oxidase (HOX, Dbv29), isoamyl alcohol oxidase (IAO) or short- and long-chain alcohol oxidases (SCAOs, LCAOs). Labelling studies proved the existence of the aldehyde hydrate as intermediate (Van Hellemond et al. 2009), and for AAO, which naturally oxidises benzylic alcohols, NMR studies revealed that the *gem*-diol intermediate was favoured (Ferreira et al. 2010) (Scheme 3).

Structurally, most of the flavoprotein oxidases either belong to the glucose-methanol-choline (GMC) oxidase or the vanillyl alcohol oxidase (VAO) family. Both families have a flavin present in the active site where the binding domain and the binding mode of the flavin differ. In case of VAO, the flavin is covalently linked to a histidine, cysteine or tyrosine residue, while in the GMC family, the majority of the enzymes contain a dissociable flavin adenine dinucleotide (FAD) moiety. In P2O or CHO, a covalent linkage was found. The active sites and consequently the substrate scope of these enzymes show high variance (Fraaije et al. 1998a; Kiess et al. 1998; Leferink et al. 2008; Dijkman et al. 2013).

**Scheme 5:**
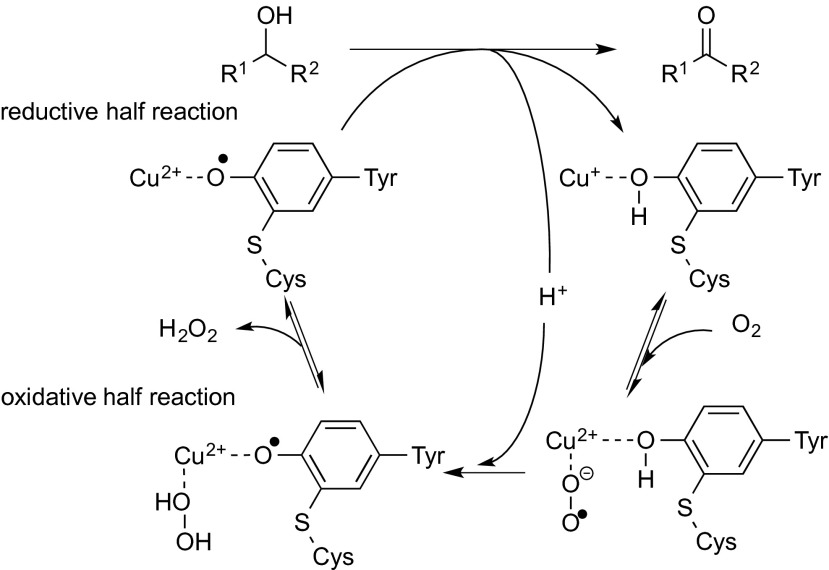
Catalytic cycle of copper-containing oxidases

Another redox cofactor found in alcohol oxidases, such as galactose oxidase (GOase), is the transition metal copper, whose role in catalysis is well described in several reviews (Ridge et al. 2008; Guengerich 2013). Since only a single copper(I) ion is found in the active site, it seems surprising that a two-electron transfer can occur. Detailed investigations revealed that the latter proceeds via two consecutive single-electron transfer steps. Thus, abstraction of the first electron by Cu^2+^ yields Cu^+^, which transfers its electron onto a tyrosine residue, which forms a transient radical anion (Monti et al. 2011). The latter is stabilised by a rare covalent thioether bridge with an adjacent cysteine (Ito et al. 1991). The second electron transfer yields a Cu^+^-tyrosine radical. From this, oxygen accepts two electrons (Wang 1998; Whittaker 2003) (Scheme 5). GOase from *Fusarium* NRRL 2903 is the most prominent member of Cu-containing alcohol oxidases and belongs to the family of radical copper oxidases, a family with a wide phylogenetic distribution and broad range of functions. The crystal structure of the enzyme revealed that a mononuclear copper ion is centred in a distorted pyramid structure, which is coordinated by two tyrosine residues (Tyr272 and Tyr495) and two histidine side chains (His496 and His581) (Whittaker and Whittaker 2001).

For Cu-containing alcohol oxidases, the oxidation stops at the aldehyde stage and over-oxidation was not observed (Monti et al. 2011).

From a biocatalytic viewpoint, alcohol oxidases are a promising group of enzymes, because they are biochemically well characterised and a broad range of enzymes have been described (Whittaker 2003; Leferink et al. 2008; Dijkman et al. 2013) which were also employed in cascade reactions (Fuchs et al. 2012; Perez-Sanchez et al. 2013; Schrittwieser et al. 2011). Depending on their role in nature, substrates for alcohol oxidases vary to a great extent in terms of substrate size and/or polarity (Turner 2011). In fungi, extracellular alcohol oxidases produce hydrogen peroxide (needed for lignin degradation by peroxidases) by oxidation of cinnamyl alcohols (e.g. coniferyl, coumaryl and sinapyl alcohol). Furthermore, hydrogen peroxide acts as antibiotic in the rhizosphere to protect roots (Monti et al. 2011). As an alternative to alcohol oxidases, NAD(P)^+^-dependent alcohol dehydrogenases provide a well-investigated enzyme platform for the oxidation of *prim*- and *sec*-alcohol functionalities. Although these enzymes are more abundant than alcohol oxidases, the equilibrium for oxidation is strongly disfavored but can be overcome by NAD(P)^+^ recycling (Hollmann et al. 2011).

In the following, an overview on the current literature of alcohol oxidases is given, by focussing on their substrate tolerance to facilitate the choice of an appropriate enzyme for a given type of alcohol substrate.

## Non-activated alcohols

### Primary aliphatic alcohols

**Scheme 6:**
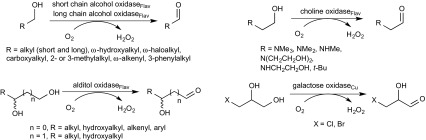
Oxidation of primary alcohols by oxidases

The enzymatic oxidation of non-activated aliphatic *prim*-alcohols by alcohol oxidases shows a remarkably broad substrate tolerance (Table 1) and encompasses straight-chain or branched substrates with chain lengths ranging from C_1_ to C_16_. In addition, functional groups, such as aromatics, halogens (Cl, Br), non-allylic olefins, alkylamino groups and carboxylates, are tolerated (Scheme 6). Vicinal, 1,3- and α,ω-diols are selectively oxidised at the *prim*-hydroxy group, while *sec*-alcohols remain untouched. Depending on the enzyme, the oxidation products are the corresponding aldehydes and carboxylic acids, which are formed by over-oxidation with flavoprotein oxidases.

**Table 1:** Primary aliphatic alcohols

Entry	Substrate	Oxidase	Reference
Mono-alcohols			
1	1-Alkanols C_1_–C_5_	SCAO^b^ from *P. pastoris* (C_1_–C_4_), *Hansenula* sp*.* (C_1_–C_5_), *C. boidinii*, *T. aurantiacus* and *A. terreus* (C_1_–C_2_)	Kato et al. 1976; Ko et al. 2005; Perez-Sanchez et al. 2013; Kumar and Goswami 2006; Siebum et al. 2006; Menon et al. 1995; Couderc and Baratti 1980; Kjellander et al. 2013
2	1-Alkanols C_7_–C_14_, C_16_	SCAO^b^ from *A. terreus* (C_7_); LCAO^b^ from *A. terreus* (C_7_–C_14_), *A. thaliana* (C_12_, C_16_) and *C. tropicalis* (C_8_, C_10_, C_12_, C_14_)	Kumar and Goswami 2006; Kumar and Goswami 2008; Kumar and Goswami 2009; Eirich et al. 2004; Cheng et al. 2004
3	2-Bromoethanol 2-Chloroethanol	SCAO from *C. boidinii*	Menon et al. 1995
Diols and triols			
4	1,2-Ethanediol	SCAO^b^ from *T. aurantiacus* and *P. pastoris*	Ko et al. 2005; Kjellander et al. 2013
5	1,10-Decanediol	LCAO^b^ from *C. tropicalis*	Eirich et al. 2004
6	1,16-Hexadecanediol	LCAO^b^ from *A. terreus*, *C. tropicalis and A. thaliana*	Kumar and Goswami 2006; Eirich et al. 2004; Cheng et al. 2004
7	1,2-Alkanediol C_4_–C_6_	AldO^b^ from *S. coelicolor*	Van Hellemond et al. 2009
8	1,3,5-Pentanetriol 1,2,4-Butanetriol	AldO^b^ from *S. coelicolor*	Van Hellemond et al. 2009
9	1,3-Butanediol	AldO^b^ from *S. coelicolor*	Van Hellemond et al. 2009
10	3-Chloro-1,2-propanediol 3-Bromo-1,2-propanediol	GOase^a^ from *Fusarium* NRRL 2903	Klibanov et al. 1982
11	1-Phenyl-1,2-ethanediol	AldO^b^ from *S. coelicolor* and *A. cellulolyticus* 11B	Van Hellemond et al. 2009; Winter et al. 2012
12	ω-Hydroxy-carboxylic acid	LCAO^b^ from *A. terreus* (C_12_) and *C. tropicalis* (C_12_, C_16_)	Kumar and Goswami 2006; Eirich et al. 2004; Cheng et al. 2004
Amino alcohols			
13	Choline *N*,*N*-Dimethyl-ethanolamine *N*-Methyl-ethanolamine Triethanolamine Diethanolamine	CHO^b^ from *A. globiformis*	Gadda et al. 2004; Ikuta et al. 1977
Unsaturated alcohols			
14	3-Buten-1-ol 4-Penten-1-ol	SCAO^b^ from *P. pastoris*	Siebum et al. 2006
15	3-Buten-1,2-diol 4-Pentene-1,2-diol	AldO^b^ from *S. coelicolor*	Van Hellemond et al. 2009
Branched alcohols			
16	2-Methyl-1-alkanol (C_4_–C_6_)	SCAO^b^ from *C. boidinii*, *P. pastoris* and *Hansenula* sp.	Clark et al. 1995
17	3-Methyl-1-butanol	SCAO^b^ from *T. aurantiacus*; SAO^c^ from *A. terreus*; IAO^b^ from *A. oryzae*	Ko et al. 2005; Eirich et al. 2004; Yamashita et al. 1999
18	1-Phenyl-3-propanol	SCAO^b^ from *A. terreus*	Kumar and Goswami 2009
19	3,3-Dimethylbutan-1-ol	CHO^b^ from *A. globiformis*	Gadda et al. 2004

^a^Copper containing

^b^Flavin containing

^c^None heme Fe^2+^ containing

Aliphatic alcohols with a chain length of one to seven C atoms were oxidised by short-chain alcohol oxidases (SCAOs) [EC 1.1.3.13] from *Pichia pastoris*, *Hansenula* sp. (Table 1, entry 1) and *Aspergillus terreus* (Table 1, entry 2) while methanol and ethanol were also converted by alcohol oxidases from *Candida boidinii*, *Thermoascus aurantiacus* and *A. terreus* (Table 1, entry 1) (Kato et al. 1976; Siebum et al. 2006; Menon et al. 1995; Couderc and Baratti 1980; Kumar and Goswami 2006; Ko et al. 2005; Perez-Sanchez et al. 2013). In general, the activity decreases with increasing chain length of the fatty alcohol, e.g. 1-pentanol shows 24 % relative activity compared to methanol (Ko et al. 2005). SCAO from *Hansenula* sp. was employed together with a C–C lyase in a cascade reaction, where short-chain alcohols (methanol, ethanol, 1-propanol and 1-pentanol) were oxidised with excellent conversion to the corresponding aldehydes, which were subjected to cross-acyloin condensation with benzoin generated in situ from benzaldehyde by benzaldehyde lyase to yield 2-hydroxyketones (Shanmuganathan et al. 2012; Perez-Sanchez et al. 2013). *prim*-Alcohols with a chain length of 7 to 16 carbon atoms were best oxidised by long-chain alcohol oxidases (LCAOs) [EC 1.1.3.20] from *A. terreus*, *Candida tropicalis* and *Arabidopsis thaliana* (Table 1, entry 2). Both, SCAOs and LCAOs, are flavoproteins located in fungal microsomes (Kemp et al. 1988; Eirich et al. 2004; Kumar and Goswami 2006, Cheng et al. 2004). Terminal alcohols bearing a polar functional group, such as α,ω-diols (Table 1, entries 5 and 6) and ω-carboxy fatty alcohols (Table 1, entry 12), with a long hydrocarbon backbone were also oxidised by long-chain alcohol oxidases (Kumar and Goswami 2006).

Short-chain alcohol oxidase from several microorganisms (*C. boidinii*, *Hansenula* sp., *P. pastoris and T. aurantiacus*) was described to convert racemic branched alcohols (Table 1, entries 16–17) in an enantioselective fashion with conversions of 16–76 %, the non-reacted substrate enantiomers showed *ee*s of up to 90 % for SCAO from *C. boidinii* (Clark et al. 1995). Isoamyl alcohol oxidase (IAO) [EC 1.1.3.x] from *Aspergillus oryzae* exhibits a narrow substrate range and prefers branched short-chain alcohols, such as 3-methyl-1-butanol (Table 1, entry 17) (Yamashita et al. 1999). Halogen-substituted alcohols, which were oxidised by SCAO, were used as molecular probes for mechanistic studies (Menon et al. 1995).

Saturated and unsaturated *vic*-1,2-diols were the substrates of choice for alditol oxidase [EC 1.1.3.41] from *Streptomyces coelicolor* and *Acidothermus cellulolyticus* (Table 1, entries 7–9, 11 and 15). This enzyme apparently prefers a glycol or 1,3-diol moiety. For *rac*-1-phenyl-1,2-ethanediol carrying a bulky aryl moiety, the (*R*)-enantiomer was preferentially oxidised by alditol oxidase (Table 1, entry 11) (Van Hellemond et al. 2009). Short (non-allylic) unsaturated alcohols lacking a second hydroxy group were completely (4-penten-1-ol) or partially (3-buten-1-ol) oxidised by short-chain alcohol oxidase from *P. pastoris* (Table 1, entry 14) (Siebum et al. 2006).

Another prominent enzyme of this group is choline oxidase from *A. globiformis* which oxidises choline and analogues, such as *N*,*N*-dimethylethanolamine, *N*-methylethanolamine, triethanolamine, diethanolamine and 3,3-dimethylbutan-1-ol (Table 1, entries 13 and 19) in a two-step oxidation to the corresponding carboxylic acid (Ikuta et al. 1977; Gadda et al. 2004).

### Secondary aliphatic alcohols

**Scheme 7:**
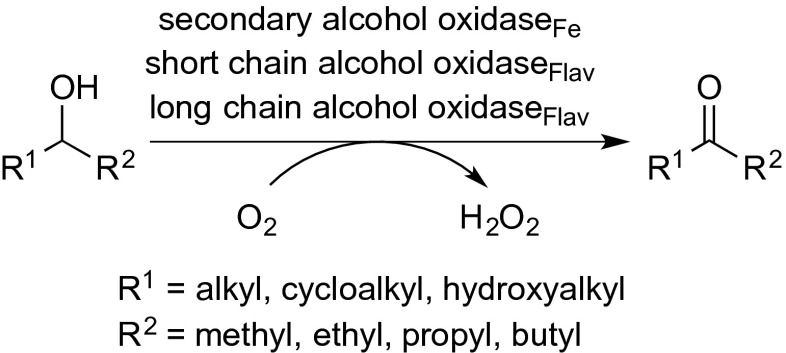
Oxidation of secondary alcohols by alcohol oxidases

Racemic secondary aliphatic alcohols are interesting substrates, because enantioselectivities in kinetic resolution are usually much higher than with *prim*-alcohols bearing a stereogenic centre. In contrast to *prim*-alcohols, which may undergo over-oxidation to carboxylic acids, the oxidation products derived from *sec*-alcohols are solely ketones (Scheme **7**). Compared to *prim*-alcohol oxidases, enzymes acting on secondary alcohols are less abundant, but several enzymes were found to be highly active (Table 2). Secondary alcohol oxidase (SAO) [EC 1.1.3.18] from *Pseudomonas putida*, *Pseudomonas vesicularis* and *A. terreus* has shown high activity for the polymeric substrate polyvinyl alcohol (PVA) (Table 2, entry 1) (Sakai et al. 1985; Kawagoshi and Fujita 1997), and it was discovered that one non-heme Fe^2+^ species is present in the enzyme. To date, it remains unclear whether the iron species serves as a cofactor like the copper in galactose oxidase or if it does not participate in catalysis at all.

**Table 2:** Secondary aliphatic alcohols

Entry	Substrate	Oxidase	Reference
1	Polyvinyl alcohol	SAO^a^ from *P. putida* and *P. vesicularis*	Sakai et al. 1985; Kawagoshi and Fujita 1997
2	2-Alkanols C_3_–C_12_, C_16_	SAO^a^ from *A. terreus* (C_3_, C_8_, C_12_), *P. putida* (C_3_–C_7_) and *P. vesicularis* (C_4_–C_8_); SCAO^b^ from *T. aurantiacus* (C_3_–C_4_), *A. terreus* (C_8_) and *P. pastoris* (C_3_); LCAO^b^ from *C. tropicalis* (C_10_–C_11_, C_16_)	Ko et al. 2005; Kumar and Goswami 2006; Kjellander et al. 2013; Sakai et al. 1985; Kawagoshi and Fujita 1997; Kumar and Goswami 2009; Eirich et al. 2004
3	3-Alkanols C_5_–C_8_	SAO^a^ from *P. putida* (C_5_–C_8_), *A. terreus* (C_8_) and *P. vesicularis* (C_6_–C_8_)	Sakai et al. 1985; Kawagoshi and Fujita 1997; Eirich et al. 2004
4	4-Alkanols C_7_–C_10_	SAO^a^ from *P. putida* (C_7_–C_9_) and *P. vesicularis* (C_7_, C_10_)	Sakai et al. 1985; Kawagoshi and Fujita 1997
5	5-Nonanol	SAO^a^ from *P. putida*	Sakai et al. 1985
6	Cycloalkanols C_6_, C_8_	SAO^a^ from *P. vesicularis* (C_6_) and *A. terreus* (C_8_)	Kawagoshi and Fujita 1997; Kumar and Goswami 2006
7	1,2-Propanediol	SAO^a^ from *P. putida*	Sakai et al. 1985
8	2,4-Pentanediol	SAO^a^ from *P. vesicularis*	Kawagoshi and Fujita 1997

^a^Non-heme Fe^2+^ containing

^b^Flavin-containing

For monomeric *sec*-alcohols, the relative activity of SAO from *P. putida* ranges between 5 and 30 % (compared to PVA). High activity for 2-octanol was found with the enzyme from *P. vesicularis* (83 % rel. activity), which also accepts cyclohexanol (42 % rel. activity). Its oxidised product (cyclohexanone) is used as a starting material for the synthesis of the polymer building block ε-caprolactam. *sec*-Alcohols bearing an additional OH group, such as 1,2-propanediol and 2,4-pentanediol, were also accepted as substrates (Table 2, entries 7 and 8); however, no details are reported about the regioselectivity of the oxidation (Sakai et al. 1985; Kawagoshi and Fujita 1997). Additionally, SCAO from *T. aurantiacus*, *A. terreus and P. pastoris* as well as LCAO from *C. tropicalis* showed broad activity on secondary alcohols (Table 2, entry 2) (Eirich et al. 2004; Kumar and Goswami 2009; Kjellander et al. 2013; Ko et al. 2005). Furthermore, 2-methyl-2-propanol was claimed to show 16 % relative activity with SCAO, but this *tert*-alcohol should be a non-substrate (Ko et al. 2005).

## Activated alcohols

### Allylic alcohols

**Scheme 8:**
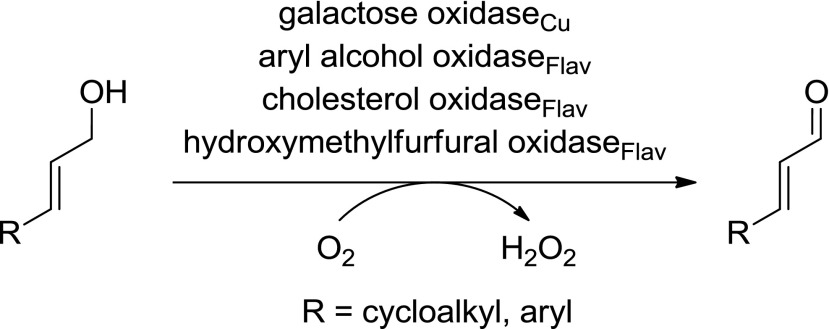
Oxidation of allylic alcohols by alcohol oxidases

In contrast to saturated (non-activated) aliphatic alcohols, allylic and benzylic alcohols are much easier to oxidise, because radicals and carbene ions occurring as intermediates are resonance stabilised (see Electronic Supplementary Material, Scheme S1). Owing to their high intrinsic reactivity, allylic alcohols are easily oxidised by a broad range of alcohol oxidases, such as copper-containing galactose oxidase (GOase) [EC 1.1.3.9], flavoprotein cholesterol oxidase (ChOx) [EC 1.1.3.6], aryl alcohol oxidase (AAO) [EC 1.1.3.7] and hydroxymethylfurfural oxidase (HMFO) [EC 1.1.3.47] (Scheme 8, Table 3) (Guillen et al. 1992; Dieth et al. 1995; Sun et al. 2002; Dijkman and Fraaije 2014).

**Table 3:**
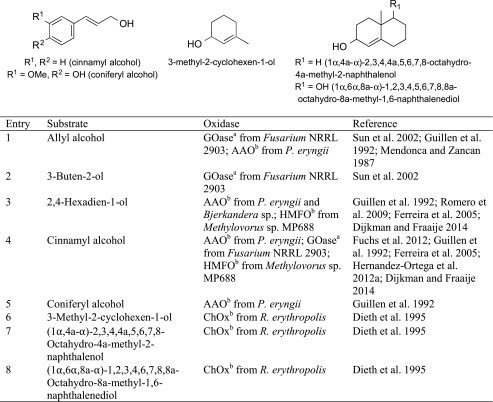
*prim*- and *sec*-Allylic alcohols

^a^Copper containing

^b^Flavin containing

Small allylic alcohol was oxidised poorly by galactose oxidase (Table 3, entries 1 and 2), which prefers large analogues, such as cinnamyl alcohol (Table 3, entry 4). A mutant of galactose oxidase from *Fusarium* sp. oxidised cinnamyl alcohol with full conversion (Sun et al. 2002; Fuchs et al. 2012). In contrast to galactose oxidase, which does not accept *sec*-allylic alcohols, cholesterol oxidase from *Rhodococcus erythropolis* converted sterically demanding secondary allylic alcohols in a complete stereo- and enantioselective fashion with conversions up to 70 % and high to excellent *ee*s. For methyl-substituted bicyclic substrates (Table 3, entries 7 and 8), the relative (*cis*) position of the hydroxyl group with respect to the methyl group were mandatory to be accepted and non-activated (non-allylic) hydroxy groups were unreactive. Even comparably small monocyclic substrates could be converted (Dieth et al. 1995). Aryl alcohol oxidase exhibits a broad substrate scope and accepts phenyl substituted allylic alcohols such as coniferyl and cinnamyl alcohol (Table 3, entries 4 and 5), as well as slim counterparts, such as 2,4-hexadien-1-ol (Table 3, entry 3), which shows that this enzyme does not necessarily need a cyclic structure, but only a conjugated system (Ferreira et al. 2005; Romero et al. 2009). 5-Hydroxymethylfurfural oxidase exhibited a similar behaviour and appears to be a promising candidate for the oxidation of allylic alcohols, as it showed excellent acceptance of cinnamyl alcohol (Table 3, entry 4) and 2,4-hexadien-1-ol (Table 3, entry 3) (Dijkman and Fraaije 2014). With cinnamyl alcohol and its *p*-methoxy derivative, AAO shows over-oxidation and forms the corresponding acids (Table 3, entry 4) (Guillen et al. 1992).

### Benzylic alcohols

**Scheme 9:**
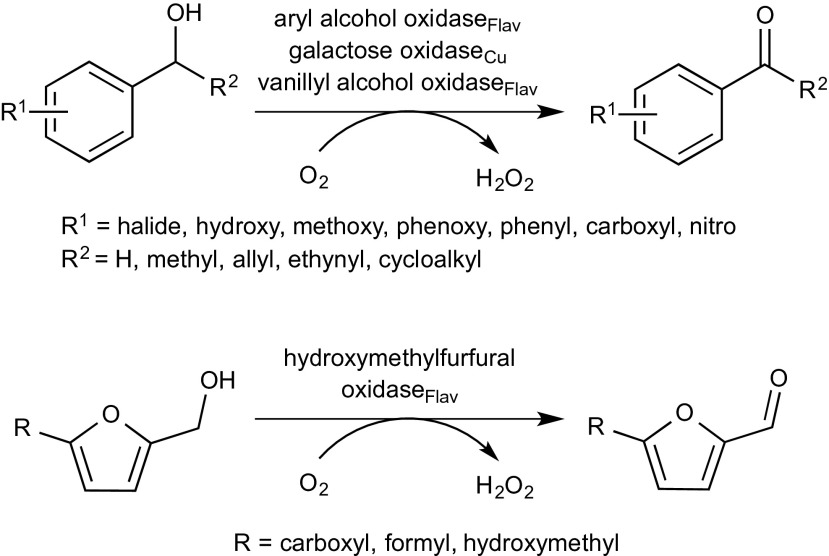
Oxidation of benzylic alcohols by alcohol oxidases

Due to their high degree of electronic activation, benzylic alcohols (Scheme 9, Table 4) are easy to oxidise. In particular, galactose oxidase from *Fusarium* NRRL 2903 and aryl alcohol oxidase from *Pleurotus eryngii* are ideally suited for this substrate type, together with a recently discovered flavin-containing oxidase from *Bjerkandera* sp.

**Table 4:**
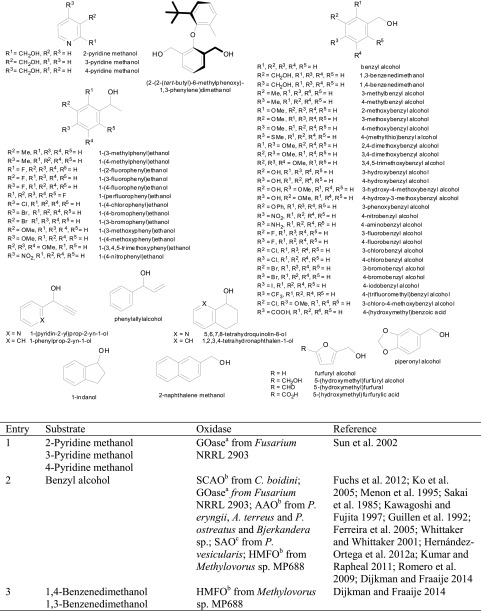

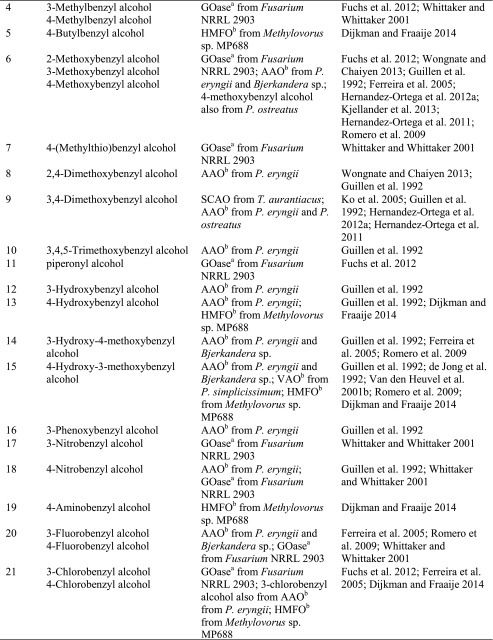

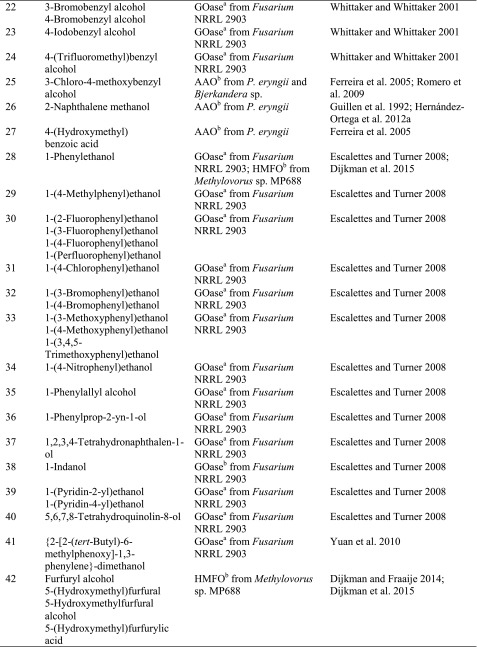
*prim*- and *sec*-Benzylic alcohols

^a^Copper containing

^b^Flavin containing

^c^Fe^2+^ containing

In the case of benzyl alcohol, two more AAOs (from *A. terreus* and *Pleurotus ostreatus*) showed activity, as well as SCAO from *C. boidinii* and *T. aurantiacus*, SAO from *P. vesicularis* and HMFO from *Methylovorus* sp. (Table 4, entry 2) (Kumar and Rapheal 2011). Although not *visible* on a non-chiral substrate, AAO acts in a stereoselective fashion by removing the pro-*R* hydride as shown by deuterium experiments (Hernandez-Ortega et al. 2012b). Various substituents on the aromatic ring system are freely tolerated: Although wild-type galactose oxidase from *Fusarium* has a broad substrate scope for benzylic *prim*-alcohols, the activity was considerably increased by mutations. For instance, all regioisomers of pyridine methanol were transformed by a R330K, Q406T-mutant of galactose oxidase, which showed up to 2000-fold enhanced activity towards 2-pyridine methanol compared to the canonical d-galactose (Sun et al. 2002). *Meta*- and *para*-substituted substrates (3-F, 3-Br, 3-Cl, 3-NO_2_, 4-F, 4-Cl, 4-Br, 4-I, 4-NO_2_, 4-OMe, 4-SMe, 4-Me, 4-CF_3_) (Table 4, entries 4, 6, 7, 17, 18, 20–24) were converted with up to 20-fold variation of relative rates (Whittaker and Whittaker 2001).

Secondary aryl alcohols undergo kinetic resolution with partly excellent *ee*s using an (*R*)-selective mutant of galactose oxidase from *Fusarium* sp. created by directed evolution (Table 4, entries 28–40) (Escalettes and Turner 2008). The same group also reported a rare example of the successful recognition of an atropisomeric pair of enantiomers possessing axial chirality (Table 4, entry 41) (Yuan et al. 2010). Furthermore, an engineered variant of HMFO was able to oxidise phenylethanol in a stereoselective fashion (Dijkman et al. 2015).

Methoxy groups (Table 4, entry 6) were accepted independently from the position on the ring with comparable activities relative to unsubstituted benzyl alcohol, whereas *para*-substituted analogues reacted more than fivefold faster with aryl alcohol oxidase. Furthermore, dimethoxy benzyl alcohols (Table 4, entries 8 and 9) were converted by aryl alcohol oxidase with high activity (Hernandez-Ortega et al. 2011; Hernandez-Ortega et al. 2012a). In particular, 3,4-dimethoxybenzyl alcohol (veratryl alcohol, Table 4, entry 9) was converted with 326 % activity, while the 2,4-substituted pendant (Table 4, entry 8) was accepted with 178 % activity relative to benzyl alcohol (Guillen et al. 1992). Sterically demanding 3,4,5-trimethoxybenzyl alcohol (Table 4, entry 10) was converted slowly. Besides methoxy groups, also hydroxy groups, combinations thereof and even a *meta*-substituted phenoxy group were accepted (Table 4, entries 12–16). The hydroxy substrates (Table 4, entries 12 and 13) were poorly converted compared to the 3-phenoxybenzyl alcohol (Table 4, entry 16) which was well accepted (Guillen et al. 1992). Additionally, the name-giving enzyme for the VAO family, vanillyl alcohol oxidase (VAO) [EC1.1.3.38] acts on 4-hydroxy-3-methoxybenzyl alcohol (vanillyl alcohol, Table 4, entry 15) (de Jong et al. 1992; Van den Heuvel et al. 1998; Fraaije et al. 1998b; Van Den Heuvel et al. 2000; Van den Heuvel et al. 2001a; Van den Heuvel et al. 2001b). While the enzyme seems to accept bulky substituents, e.g. bearing a phenoxy group, additional methoxy or especially hydroxy groups (Table 4, entries 12–16) cause unfavourable interactions in the active site. The aryl alcohol oxidase from *P. eryngii* also acts on 4-hydroxy-substituted α-aryl alcohols (Table 4, entry 13) (Guillen et al. 1992). Piperonyl alcohol (1,3-benzodioxole-5-methanol, Table 4, entry 11), a building block in epinephrine synthesis, was oxidised with full conversion by galactose oxidase from *Fusarium* sp. (Fuchs et al. 2012). A broad range of chloro- and fluoro-substituted aryl alcohols were accepted by both aryl alcohol oxidase and galactose oxidase (Table 4, entries 20 and 21) (Guillen et al. 1992; Whittaker and Whittaker 2001; Romero et al. 2009). The only exception being *meta*-chlorobenzyl alcohol, which was not converted at all. A substrate which is sterically demanding and well accepted by AAO is 2-naphthalene methanol (Table 4, entry 26). It showed a relative activity of 746 % compared to the monocyclic substrate analogue (Table 4, entry 2). In conclusion, the position of substituents and their polarity seem to play a crucial role in substrate acceptance. The recently characterised 5-hydroxymethylfurfural oxidase from *Methylovorus* sp. MP688 showed a broad substrate acceptance of various furfuryl alcohols (Table 4, entry 42), but it also showed activity on benzylic alcohols with substituents in *para-*position (Table 4, entries 3 and 5) and vanillyl alcohol (Table 4, entry 15) (Dijkman and Fraaije 2014). In view of the growing importance of furan derivatives, such as hydroxymethyl furfural, which can easily be obtained via double elimination of H_2_O from hexoses or pentoses and hence constitute a promising C source for organic synthesis (Schwartz et al. 2014), HMFO has a considerable potential to be used in large-scale applications. In a recent study, site-directed mutagenesis allowed to boost the activity of HMFO on 5-formyl-2-furancarboxylic acid leading to improved yields of 2,5-furandicarboxylic acid, which is a promising monomer for polyester production from renewable resources (Dijkman et al. 2015).

## α-Hydroxy acids

**Scheme 10:**
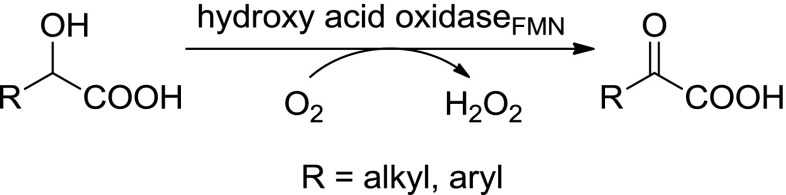
Enzymatic oxidation of hydroxy acids by hydroxy acid oxidases

Owing to the negative charge of α-hydroxy acids at neutral pH, the latter are oxidised by a subgroup of flavoprotein oxidases, which are specific for this type of polar substrate and furnish the corresponding α-ketoacids (Scheme 10). On a first glimpse, this transformation appears to have little value, because it goes in hand with the destruction of a chiral centre. However, α-hydroxy acids are usually more easily accessible than the corresponding sensitive α-keto-analogues, which are prone to decarboxylation; this transformation is of practical value, and in addition, racemic α-hydroxy acids undergo kinetic resolution with a preference for the (*S*)-enantiomer (Turner 2011).

A broad range of α-hydroxy acids were studied as substrates for FMN-depending glycolate oxidase (GlyO), l-lactate oxidase (LLO) or long-chain 2-hydroxyacid oxidase (LHAO), which belongs to the group of (*S*)-2-hydroxy acid oxidases (HAOX) [EC 1.1.3.15]. For GlyO, the natural substrate is glycolic acid (Table 5, entry 1) and the most prominent GlyO originates from spinach (*Spinacia oleracea*) (Zelitch and Ochoa 1953). The name-giving substrate can be over-oxidised to oxalic acid, although the second step is less efficient (Richardson and Tolbert 1961). Short- and medium-chain 2-hydroxy acids with up to ten carbon atoms (Table 5, entries 10 and 11), unsaturated *cis*- and *trans*-2-hydroxydec-4-enoic acid (Table 5, entry 14), bulky phenyllactic acid (Table 5, entry 9) and the oxa-analogue 2-hydroxy-4-pentoxybutyric acid (Table 4, entry 13) were well accepted as substrates in kinetic resolution with good to excellent *ee*s. Furthermore, 3-chlorolactic acid (110 % rel. activity), 2-hydroxybutanoic acid (120 % rel. activity), 3-indolelactic acid (18 % rel. activity) (Table 5, entries 9 and 10), 3,3,3-trifluorolactic acid (11 % rel. activity) (Table 5, entry 15) and 2-hydroxydecanoic acid (40 % rel. activity) (Table 4, entry 12) were nicely converted relative to lactic acid (Adam et al. 1997; Adam et al. 1998; Das et al. 2009; Stenberg et al. 1995). The substrate spectrum of FMN-containing l-lactate oxidase from *Aerococcus viridans* and a mutant thereof encompasses also sterically demanding α-hydroxy acids, such as *para*-substituted mandelic acid derivatives (Table 5, entries 5–7) and was analysed in a quantitative structure analysis (Duncan et al. 1989; Maeda-Yorita et al. 1995; Yorita et al. 1997). Additionally, an enzyme originating from *Pseudomonas stutzeri* was used to oxidise lactic acid to pyruvate enantioselectively (Table 5, entry 2, Gao et al. 2009). LHAO, on the contrary, originating from mammalian sources, such as pig kidney, rat kidney or hog renal cortex, oxidises 2-hydroxy acids with a carbon chain length of at least three C atoms (Blanchard et al. 1946; Robinson et al. 1962). 2-Hydroxy-4-methylpentanoic acid, 2-hydroxybutyric acid and also mandelic acid (Table 5, entries 8–10, 12) were oxidised with moderate conversions (Urban et al. 1988).

**Table 5:**
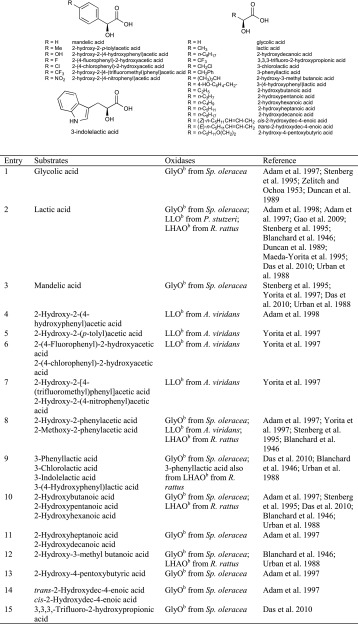
α-Hydroxy acids

^a^Copper containing

^b^Flavin containing

## Sterols

**Scheme 11:**
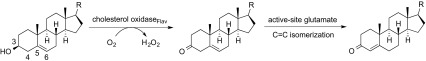
Enzymatic oxidation and C=C isomerisation of cholesterol derivatives by cholesterol oxidase

The bioactivity of steroids strongly depends on their substitutional pattern, which is dominated by secondary hydroxy groups in α- or β-positions, which upon oxidation furnish keto-steroids. This transformation can be achieved in a regio- and stereoselective fashion by alcohol oxidases. Owing to the spacious molecular framework, it is conceivable that alcohol oxidases acting on steroids have a strong preference for large substrates and are generally not ideally suited for small alcohols (Scheme 11).

Cholesterol oxidase (ChOx) [EC 1.1.3.6] found in *Streptomyces hygroscopicus*, *Rhodococcus* and *Brevibacterium sterolicum* is the enzyme of choice for the oxidation of the secondary alcohol function at C_3_, which leads to rare keto-steroids (Table 6). From a biochemical point of view, it is remarkable that cholesterol oxidases are strictly FAD containing, although they belong to two different families: Cholesterol oxidase from *Streptomyces* is a member of the GMC oxidase family, whereas *B. sterolicum* ChOx belongs to the VAO family. Remarkably, most cholesterol oxidases are bifunctional enzymes (Pollegioni et al. 1999; Gadda et al. 1997; Pollegioni et al. 2009; Vrielink and Ghisla 2009), as they not only oxidise the alcohol functionality at C_3_ yielding 5-cholesten-3-one but also mediate the isomerisation of the C_5_–C_6_ double bond of the latter into conjugation with the newly formed keto-function by assistance of an active-site glutamate residue to furnish the corresponding 4-en-3-one, as demonstrated in detail with ChOx from *B. sterolicum* (Kass and Sampson 1995) (Scheme 11). The enzyme exhibited a surprisingly broad substrate scope, and a variant from *R. erythropolis* even lacks enantiospecificity at the C_3_ position (Dieth et al. 1995; Biellmann 2001). For the enzyme from *Rhodococcus* sp., moderate activities (relative to the natural substrate cholesterol) on β-sitosterol (80 % rel. activity) and stigmasterol (78 % rel. activity) were found by Wang et al. (2008) (Table 6, entries 6 and 7). Furthermore, the enzyme was active on cholestanol, 7-dehydrocholesterol and dehydroepiandrosterone (15–37 % rel. activity) (Table 6, entries 2, 4 and 8), and 5 % relative activity was found on 5α-androstane-3α,17β-diol (Table 6, entry 11) (Labaree et al. 1997; Toyama et al. 2002; Wang et al. 2008, Fujishiro et al. 2002; Xiang and Sampson 2004). Moreover, cholesterol oxidase from *B. sterolicum* was employed for the oxidation of 7α- and 7β-hydroxycholesterol (90 % conv.) (Table 6, entry 3) in a chemoenzymatic multistep synthesis (Alexander and Fisher 1995).

**Table 6:**
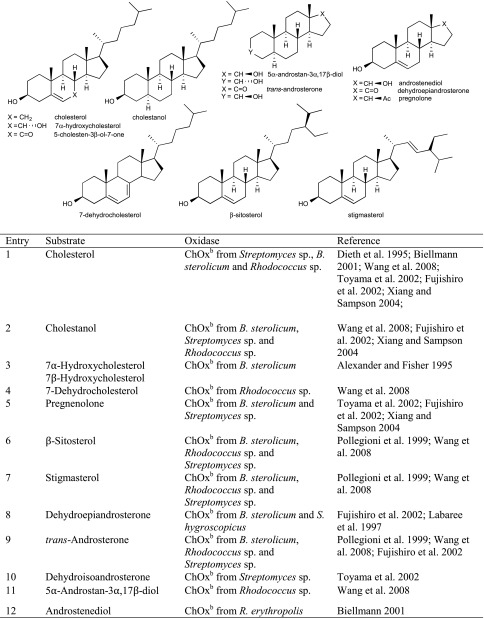
Sterols

^a^Copper containing

^b^Flavin containing

## Sugar-related alcohols

### Sugars

**Scheme 12:**
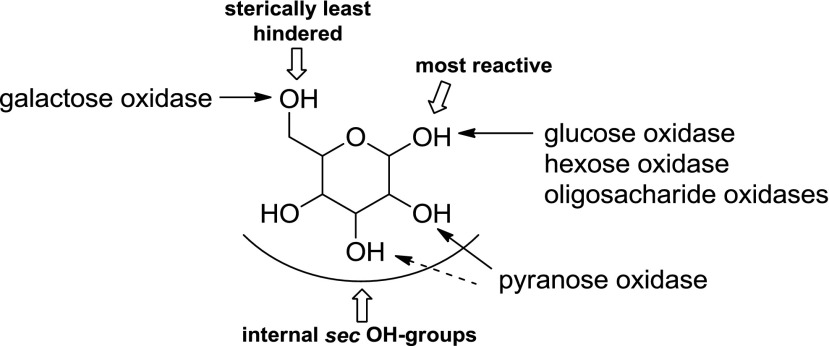
Regioselectivity of alcohol oxidases on a hexose framework

Although sugars constitute the most abundant group of renewable compounds/materials (Straathof 2014), their polyhydroxy structure imposes several unsolved problems in view of their utility as starting materials in organic synthesis: (i) they possess only a single type of functional group—the hydroxy group, and (ii) there are too many of them with similar reactivity (Scheme 12). This causes a selectivity problem, which is usually circumvented by tedious and inefficient protection-deprotection chemistry. (iii) Furthermore, except for the anomeric carbon, the carbon framework is inaccessible to C–C extension/modification, because the [CH–OH] moiety cannot be directly accessed without prior activation of the hydroxy group. In this context, regioselective oxidation of OH groups in sugars at the expense of O_2_ offers an elegant method to introduce a carbonyl group, which is an ideal acceptor for C nucleophiles in C–C bond forming reactions.

Due to the presence of numerous hydroxy groups, carbohydrates are usually bound in the active site of proteins via a tight hydrogen-bonding network, which is not possible for lipophilic mono-alcohols or diols. Consequently, one might surmise, that alcohol oxidases acting on lipophilic (mono) alcohols would not accept polar carbohydrates, and vice versa. However, comparison of Tables 1 and 3 shows that many sugar alcohol oxidases are also surprisingly active on small non-polar alcohols, in particular galactose oxidase and alditol oxidase.

The relative reactivity of hydroxy groups in sugars can be associated with different subgroups of alcohol oxidases, most of which possess a strong regio-preference for a specific hydroxyl group, which is exemplified on a schematic hexose (Scheme 12). With its hemiacetal structure, the anomeric OH is most reactive, which can be oxidised by glucose oxidase (GOX), hexose oxidase (HOX) and oligosaccharide oxidases forming the corresponding sugar lactone. Next, the terminal *prim*-OH is sterically least hindered among the non-activated hydroxy groups; it can be selectively oxidised by GOase to yield the aldehyde; no over-oxidation to the acid is observed in this case. Due to small steric and electronic differences, internal secondary hydroxy groups show very similar reactivities, they are oxidised by P2O with mixed regioselectivities with a prevalence of C_2_ > C_3_ yielding ketoses. C_3_-Oxidation products are only formed on 2-deoxy and methylated sugars.

(i) The most reactive anomeric hydroxy group in sugars can be selectively oxidised by a range of well-studied oxidases (Scheme 12): d-Glucose (Table 7, entry 1) is the natural substrate of the flavoenzyme GOX [EC 1.1.3.4], well studied from *Aspergillus niger*, which displayed a very narrow substrate spectrum and oxidises glucose at the C_1_ position (Nakamura and Ogura 1968). Furthermore, chitooligosaccharide oxidase (ChitO) [EC 1.1.3.x] from *Fusarium graminearum* catalyses the oxidation of C_1_ of d-glucose. The catalytic activity was improved by mutation (Heuts et al. 2007a), and the wild-type and mutant enzymes also accepted cellulose degradation products like cellobiose, cellotriose and cellotetraose (Table 7, entry 18). Mutants of chitooligosaccharide oxidase also accepted d-lactose and d-maltose besides the before mentioned d-glucose oligomers (Table 7, entries 9 and 10) (Heuts et al. 2007a). Variants obtained by further mutagenesis studies showed a switch in the preference for the oligosugar preference as well as improved activities on d-lactose, d-maltose and d-glucose (Ferrari et al. 2015).

**Table 7:**
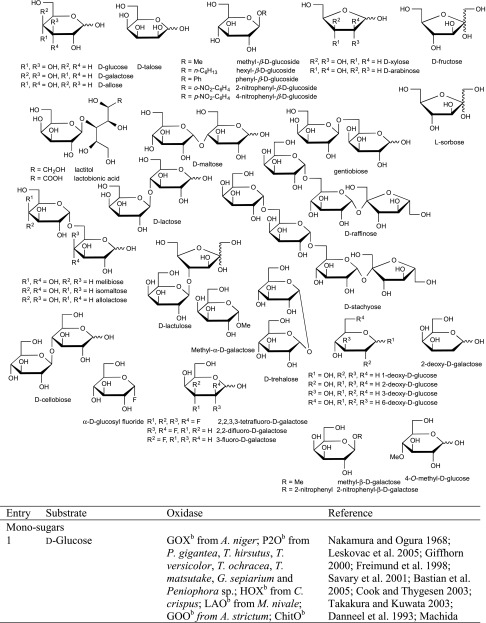

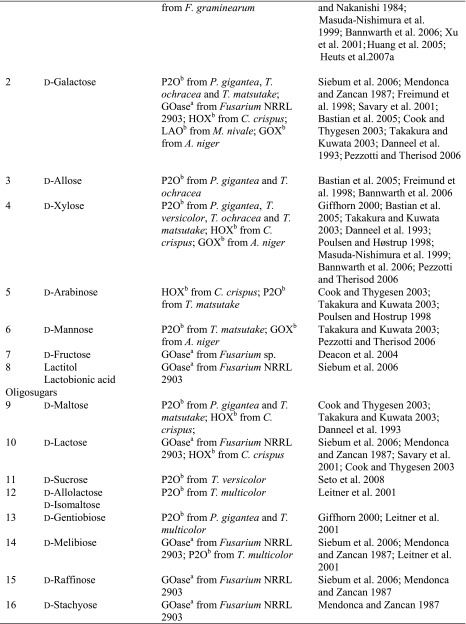

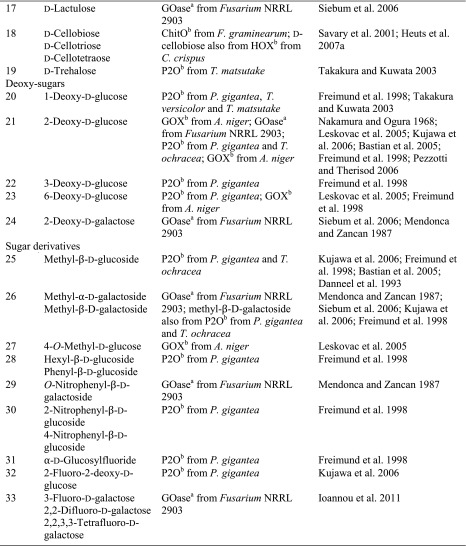

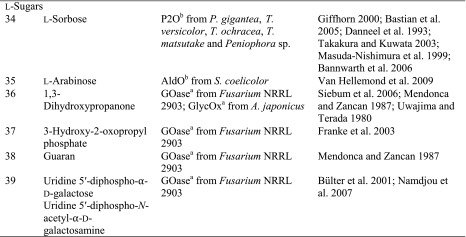
Sugars

^a^Copper containing

^b^Flavin containing

Furthermore, also glucooligosaccharide oxidase (GOO) [EC 1.1.3.x] from various sources oxidised d-glucose and its oligomers at C_1_ (Huang et al. 2005). Lactose oxidase (LAO) [EC 1.1.3.x] from *Microdochium nivale* displayed a similar substrate preference. Cellobiose (Table 7, entry 18) with 100 % relative activity was the preferred substrate, whereas di-sugars as d-maltose (84 % rel. activity) and d-lactose (52 % rel. activity) were also well accepted (Table 7, entries 9,10). Furthermore, the monosugars d-glucose (69 % rel. activity) and d-galactose (31 % rel. activity) were both oxidised at C_1_ (Xu et al. 2001) (Table 7, entries 1 and 2). Moreover, Pezzotti and Therisod synthesised aldonic acids starting with C_6_ sugars (d-galactose, d-xylose, d-mannose and 2-deoxy-d-glucose) employing glucose oxidase for the oxidation of the C_1_ hydroxy group (2006). HOX [EC 1.1.3.5] from *Chondrus crispus* is an enzyme with a fairly broad substrate scope for the oxidation of sugars at C_1_. Hexose oxidase accepted d-xylose, d-arabinose and d-glucose containing di-sugars, like d-lactose and d-cellobiose (Table 7, entries 4, 5, 10 and 18) (Poulsen and Hostrup 1998; Savary et al. 2001; Rand et al. 2006).

(ii) The sterically least hindered *prim*-OH group of sugars can be selectively oxidised by copper-containing galactose oxidase (Scheme 12). Relative activities were measured in relation to the reactivity of the C_6_-hydroxy group of d-galactose as the canonical substrate. The most prominent galactose oxidase from *Fusarium* converted d-galactose containing substrates d-lactose (10 % conv.), lactitol (20 % conv.), lactobionic acid and the synthetic disaccharide and laxativum d-lactulose completely (Table 7, entries 8, 10 and 17) (Siebum et al. 2006). For substrate acceptance of GOase, the axial position of the C_4_ position is crucial. The di-sugars d-melibiose, d-raffinose and d-stachyose were good substrates for galactose oxidase (83 % rel. activity for d-melibiose, up to 161 % rel. activity for d-stachyose) (Table 7, entries 14–16) (Mendonca and Zancan 1987). For d-fructose (Table 7, entry 7), a GOase mutant from *Fusarium* seems to be an appropriate biocatalyst (Deacon et al. 2004). Recently, a FAD-containing hexose oxidase was discovered. The so-called Dbv29 oxidised a glycopeptide at C_6_ to the corresponding carboxylic acid in a two-step reaction (Li et al. 2007; Liu et al. 2011).

(iii) d-Glucose (Table 7, entry 1) was also oxidised by the flavoenzyme pyranose oxidase (P2O) [EC 1.1.3.10] (Giffhorn 2000), which was obtained from several fungi (*Peniophora sp., Trametes sp., Tricholoma matsutake and Gloeophyllum sepiarium*). It oxidises hydroxyl groups on the C_2_ position, but also oxidation at C_3_ can occur (Scheme 12) (Kujawa et al. 2006). The process based on C_2_ oxidation of d-glucose followed by catalytic hydrogenation yielding d-fructose is known as ‘Cetus process’, which was also utilised for the synthesis of d-tagatose (Geigert et al. 1983; Freimund et al. 1996). d-Galactose was a rather poor substrate for pyranose oxidase from *P. gigantea* (Table 7, entry 2) (Freimund et al. 1998; Cook and Thygesen 2003; Bastian et al. 2005). Furthermore, the configuration on C_4_ played an important role in substrate acceptance. d-Allose (94 % overall yield), d-xylose (100 % overall yield) and d-mannose (only moderate rel. activity of 23 %) were all oxidised by pyranose oxidase originating from several microorganisms (Table 7, entries 3, 4 and 6) (Danneel et al. 1993; Freimund et al. 1998; Takakura and Kuwata 2003; Bannwarth et al. 2006; Machida and Nakanishi 1984). Pyranose oxidase accepted the di-sugars d-trehalose (54 % rel. activity), d-gentiobiose (1 % conversion) and d-maltose (8–56 % rel. activity) as substrates (Table 7, entries 9, 13 and 19) (Danneel et al. 1993; Freimund et al. 1998; Takakura and Kuwata 2003). Moreover, P2O was used as a biocatalyst for the C_2_ oxidation of disaccharides to obtain 2-keto-aldopyranose intermediates (Leitner et al. 2001) and the di-sugar d-sucrose (Table 7, entry 11) was fully converted by P2O in a multistep process (Seto et al. 2008).

Deoxy sugars were often employed in kinetic studies to investigate the catalytic mechanism of enzymes. 1-, 2-, 3- and 6-deoxy-d-glucose and 2-deoxy-d-galactose (Table 7, entries 20–24) were used for this purpose showing full conversions. The enzymes exhibited their expected regioselectivity. For pyranose oxidase, activity was observed for 2-deoxy-d-glucose (52 % rel. activity) for oxidation at C_3_ (Table 7, entry 21). 1-Deoxy-d-glucose (Table 7, entry 20) was converted by pyranose oxidase (8 % rel. conversion P2O from *Phanerochaete gigantea*, 22 % from *Trametes versicolor* and 69 % from *T. matsutake*). The substrate 3-deoxy-d-glucose was almost as good for pyranose oxidase as the natural one, but 6-deoxy-d-glucose showed significantly diminished relative conversion rate of 15 % (Table 7, entries 22 and 23). Glucose oxidase also shows activity for 2-deoxy-d-glucose and 6-deoxy-d-glucose (Table 7, entries 21 and 23). Galactose oxidase showed 74 % relative activity for 2-deoxy-d-galactose (Table 7, entry 24) (Danneel et al. 1993; Freimund et al. 1998; Takakura and Kuwata 2003; Leskovac et al. 2005; Siebum et al. 2006; Masuda-Nishimura et al. 1999).

In addition, various sugar derivatives were tested: 4-*O*-Methylated sugars were accepted by pyranose oxidase and galactose oxidase (Schoevaart and Kieboom 2004). With pyranose oxidase, oxidation occurred at C_3_. Phenyl- and hexyl-glucosides were well accepted, but underwent a glycosyl transfer reaction forming a disaccharide (Table 7, entry 28). These bulky substrates indicate that the size of the active site is not a limiting factor. Nitro sugars were tested with pyranose oxidase, and glycosyl transfer occurred yielding a 4:1 ratio of 1-6 vs. 1-3 di-sugar at C_2_ position in 15 % overall yield. At C_4_ position, a 2:1 mixture of 1-6 vs. 1-3 di-sugar was obtained in 24 % yield. α-d-Glucosyl fluoride (Table 7, entry 31) was a moderate substrate for pyranose oxidase from *P. gigantea* (40 % yield, Danneel et al. 1993; Freimund et al. 1998). Pyranose oxidase also converted the *unnatural*l-sugar l-sorbose completely (Table 7 entry 34). Mono- and poly-fluorinated galactose analogues were oxidised by galactose oxidase (Table 7, entry 33) (Ioannou et al. 2011), and also hydroxyacetone derivatives represented excellent substrates. Dihydroxyacetone (Table 7, entry 36) was also oxidised at a fair rate by glycerol oxidase (GlycOx) from *Aspergillus japonicus* (59 % rel. activity) (Uwajima and Terada 1980). Furthermore, galactose oxidase was active on guaran, a galactomannan (Table 7, entry 38) (47 % rel. activity) (Mendonca and Zancan 1987). This enzyme was also applied for the oxidation of the nucleotide sugars uridine 5′-diphospho-α-d-galactose and uridine 5′-diphospho-*N*-acetyl-α-d-galactosamine (Table 7, entry 39) for subsequent biotinylation (Bülter et al. 2001; Namdjou et al. 2007).

### Sugar alcohols and amino sugar alcohols

Several enzymes were reported to oxidise sugar alcohols to the corresponding aldoses, and in case of flavoprotein oxidases, aldonic acids were obtained via over-oxidation. FAD-containing alditol oxidase (AldO) [EC 1.1.3.41] has shown a broad acceptance for sugar alcohols: AldO from *Streptomyces* sp. and thermophilic *A. cellulolyticus* acted on several d- and even l-sugar alcohols (Table 8) and oxidised them to the corresponding aldoses or even further to carboxylic acids. d-Galactitol, d-xylitol, d-sorbitol, d-mannitol, l-threitol and prochiral glycerol (Table 8, entries 1–5, 9) were tested as substrates in kinetic studies (Heuts et al. 2007b; Forneris et al. 2008; Van Hellemond et al. 2009; Murooka and Yamashita 2001; Drueckhammer et al. 1991; Yamashita et al. 2000). Glycerol was oxidised to l-glyceraldehyde as a building block for a follow-up aldolase reaction in a multienzyme cascade (Franke et al. 2003). The latter is also oxidised by the Cu-containing glycerol oxidase which exhibited excellent activity towards glycerol, which was selected as a name-giving substrate (Uwajima and Terada 1980; Uwajima et al. 1984). The building block dihydroxyacetone phosphate (DHAP), which is a popular C donor in asymmetric aldol reactions, can be obtained using flavoprotein glycerol 3-phosphate oxidase (GPO) [EC 1.1.3.21] for the oxidation of l-glycerol 3-phosphate (Table 8, entry 10) at the *sec*-OH (Babich et al. 2011). Furthermore, also copper-containing galactose oxidase from *Fusarium* exhibited a broad acceptance of sugar alcohols without acid formation (Table 8, entries 1, 2, 5, 6 and 8).

**Table 8:**
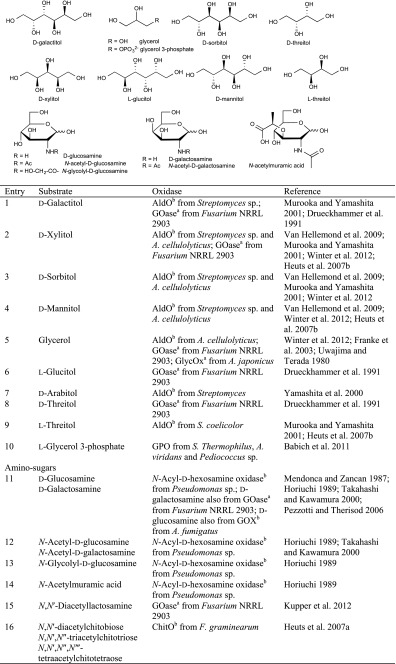
Sugar alcohols and amino sugars

^a^Copper containing

^b^Flavin containing

For the oxidation of amino sugars, *N*-acyl-d-hexosamine oxidase [EC 1.1.3.29] from *Pseudomonas* sp. is the enzyme of choice, although also galactose oxidase showed activities on this substrate class (Mendonca and Zancan 1987; Takahashi and Kawamura 2000). *N*-Acetyl-d-galactosamine (Table 8, entry 12) was converted almost as fast as the natural substrate (98–99 % rel. activity) by *N*-acyl-d-hexosamine oxidase. It seems that (in contrast to other enzymes) the configuration of C_4_ is not relevant for substrate acceptance of *N*-acyl-d-hexosamine oxidase. Amino sugars without *N*-acyl function, such as d-glucosamine (26 % rel. activity) and d-galactosamine (81 % rel. activity), were moderate substrates (Table 8, entry 11), like *N*,*N*′-diacetylchitobiose (31–49 % rel. activity) and *N*-acetylmuramic acid (44 % rel. activity) (Table 8, entry 14) with respect to the natural substrate *N*-acetyl-d-glucosamine (Horiuchi 1989; Takahashi and Kawamura 2000). The diamino sugar *N*,*N*′-diacetyllactosamine and oligomers thereof were successfully oxidised by galactose oxidase (Kupper et al. 2012). Another enzyme which was found to be active on C_1_ of *N*-acetyl-d-glucosamine and its oligomers *N*,*N*′-diacetylchitobiose, *N*,*N*′,*N*″-triacetylchitotriose and *N*,*N*′,*N*″,*N*‴-tetraacetylchitotetraose (Table 8, entry 16) is chitooligosaccharide oxidase (ChitO) (Heuts et al. 2007a) (Table 9).

**Table 9:** Cofactor presence, substrate scope and propensity for over-oxidation of alcohol oxidases

Enzyme	Cofactor	Substrate (major activities)	Over-oxidation
Alditol oxidase (AldOx)	FAD	Primary alcohols, sugar alcohols	Yes
Aryl alcohol oxidase (AAO)	FAD	Benzylic alcohols, allylic alcohols	Yes
Chitooligosaccharide oxidase (ChitO)	FAD	Sugars	No
Cholesterol oxidase (ChOx)	FAD	Sterols, allylic alcohols	No
Choline Oxidase (CHO)	FAD	Amino alcohols	Yes
Galactose oxidase (GOase)	Cu^2+^	Benzylic alcohols, sugars	No
Glucooligosaccharide oxidase (GOO)	FAD	Sugars	No
Glucose oxidase (GOX)	FAD	Sugars	No
Glycerol Oxidase (GlycOx)	Cu^2+^	Sugar alcohols	No
Glycerol 3-phosphate oxidase (GPO)	FAD	Secondary alcohols	No
Glycolate oxidase (GlyO)	FMN	α-Hydroxy acids	No
Hexose oxidase (HOX)	FAD	Sugars	No
Hydroxymethylfurfural oxidase (HMFO)	FAD	Benzylic alcohols, allylic alcohols	Yes
(*S*)-2-Hydroxy acid oxidase (HAOX)	FMN	α-Hydroxy acids	No
Isoamyl alcohol oxidase (IAO)	FAD	Branched aliphatic alcohols	Yes
L-lactate oxidase (LLO)	FMN	α-Hydroxy acids	No
Lactose oxidase (LAO)	FAD	Sugars	No
Long-chain alcohol oxidase (LCAO)	FAD	Aliphatic alcohols	No
Secondary alcohol oxidase (SAO)	Fe^2+^	Secondary aliphatic alcohols	No
Short-chain alcohol oxidase (SCAO)	FAD	Aliphatic alcohols	Yes
Pyranose oxidase (P2O)	FAD	Sugars	No
Vanillyl alcohol oxidase (VAO)	FAD	Benzylic alcohols	Yes

## Summary and outlook

The broad substrate scope coupled with high regio- and stereoselectivity makes alcohol oxidases a fantastic tool for the oxidation of primary and secondary alcohols using molecular oxygen as an alternative to traditional chemical methods. Owing to their mechanism, copper-depending oxidases selectively yield aldehydes from primary alcohols, while over-oxidation to furnish carboxylic acids may take place to a varying degree with flavin-depending oxidases. For a broad range of alcohols—non-activated *prim*- and *sec*-alcohols, activated allylic, cinnamic and benzylic alcohols, hydroxy acids, hydroxy steroids, carbohydrates and derivatives thereof—alcohol oxidases are available from various microbial sources, which are reviewed with respect to their substrate tolerance to facilitate the choice of the optimal enzyme for a given alcohol substrate.

## Electronic supplementary material

ESM 1 (PDF 32 kb)
