# Supplementary material for: The substrate tolerance of alcohol oxidases
Source: Appl Microbiol Biotechnol. 2015 Jul 8;99(16):6617–42. doi: 10.1007/s00253-015-6699-6 (PMC4513209; doi:10.1007/s00253-015-6699-6)
Supplement: Supplementary file 1 — (PDF 32 kb) [file 253_2015_6699_MOESM1_ESM.pdf]

*Appl. Microbiol. Biotechnol.*

# The Substrate Tolerance of Alcohol Oxidases

Mathias Pickl,[b] Michael Fuchs,[b] Silvia M. Glueck,[a,b] Kurt Faber[b *]

[a]*Austrian Centre of Industrial Biotechnology (ACIB GmbH), c/o*

[b]*Department of Chemistry, Organic & Bioorganic Chemistry, University of Graz, Heinrichstrasse 28, A-8010 Graz, Austria.*

## Electronic Supplementary Material

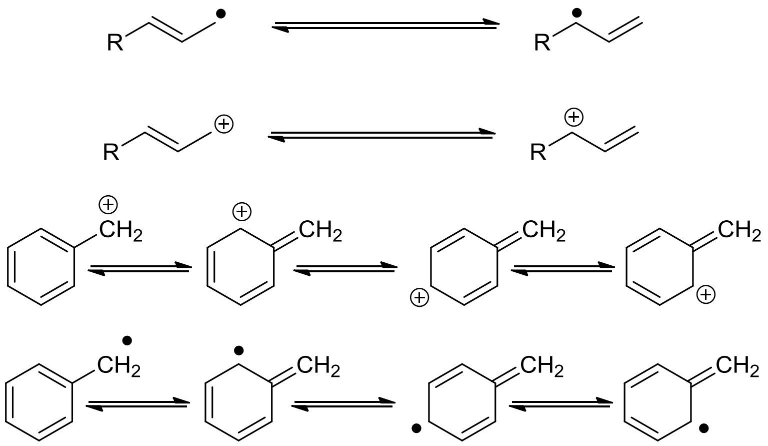


**Scheme S1:** Resonance stabilisation of allylic and benzylic species.

* Correponding author: phone +43-316-380-5332; fax: +43-316-380-9840; <Kurt.Faber@Uni-Graz.at>
